# Quantitative relations between the eyeball, the optic nerve, and the optic canal important for intracranial pressure monitoring

**DOI:** 10.1186/1746-160X-10-32

**Published:** 2014-08-17

**Authors:** Michael Vaiman, Paul Gottlieb, Inessa Bekerman

**Affiliations:** 1 Department of Otolaryngology, Head and Neck Surgery, Assaf Harofe Medical Center, Affiliated to Sackler Faculty of Medicine, Tel Aviv University, Zerifin, Israel; 2 Department of Radiology, Assaf Harofe Medical Center, Affiliated to Sackler Faculty of Medicine, Tel Aviv University, Zerifin, Israel; 3 33 Shapiro Street, Bat Yam 59561, Israel

**Keywords:** Optic nerve sheath diameter, Computed tomography

## Abstract

**Objective:**

To find correlations between diameters of the optic nerve sheath (ONSD), the eyeball, and the optic canal that might be important for intracranial pressure monitoring.

**Methods:**

In a prospective cohort study, the CT data of consecutive 400 adults (18+) with healthy eyes and optic nerves and absence of neurological diseases were collected and analyzed. When the CT scans were obtained, the diameters of the optic nerve sheath, the eyeball, and the optic canal were measured and statistically analyzed. The data obtained from the left and from the right eyeballs and optic nerves were compared. The correlation analysis was performed within these variables, with the gender, and the age.

**Results:**

In healthy persons, the ONSD varies from 3.65 mm to 5.17 mm in different locations within the intraorbital space with no significant difference between sexes and age groups. There is a strong correlation between the eyeball transverse diameter (ETD) and ONSD that can be presented as ONSD/ETD index. In healthy subjects, the ONSD/ETD index equals 0.19.

**Conclusion:**

The calculation of an index when ONSD is divided by the ETD of the eyeball presents precise normative database for ONSD intracranial pressure measurement technique. When the ONSD is measured for intracranial pressure monitoring, the most stable results can be obtained if the diameter is measured 10 mm from the globe. These data might serve as a normative database at emergency departments and in general neurological practice.

## Introduction

Intracranial pressure monitoring by means of measuring changes in the optic nerve sheath' diameter (ONSD) became practical in the 1990s. It was postulated that the presence of enlarged optic nerve sheaths suggests that raised intracranial pressure is transmitted intraorbitally [1-3]. While this fact is already well established and its importance is understood, some disagreement remains in its quantitative part. The ONSD is measured by sonography, CT, and MRI but no generally accepted protocol was designed. Different authors indicated normal/abnormal threshold (a cutoff value) of the ONSD from 5 mm to 5.9 mm with numerous variations between these numbers [4,5]. The recent review on methods of intracranial pressure monitoring estimated the accuracy of the ONSD method as low [6].

Numerous publications on the topic [1-5,7,8], actually all of them, report measurements of the ONSD only and do not take into account variations of forms and sizes of the eyeball and the optic canal as like the intraorbital part of the optic nerve is located not between these two anatomical structures but in the open space. There is a possibility that dimensions of these two structures might correlate with the ONSD influencing the accuracy of the ONSD method of intracranial pressure monitoring. If the ONSD is used as a technique for intracranial pressure monitoring, various additional factors are to be taken into account.

First, the eyeball is a constantly voluntary and involuntary moving object even when at rest and the head of the optic nerve moves with it [9]. For example, if at the moment of the image taking a patient will gaze 3 mm above the horizontal line, the distal part of the optic nerve will move 3 mm below the horizontal line; and if fixational eye movements will turn the eye 3 mm to the left, the optic nerve head will move 3 mm to the right. All that movements might change the ONSD close to the globe. The researches ignore this possibility and they usually measure the ONSD only 3 mm behind the globe [1-5,7,8]. The authors have chosen this location because the sheath is wide in this area (bulging dura mater region).

Second, the axial length of the eyeball (anterior-to-posterior diameter) is different in cases with myopia, emmetropia, and hypermetropia [10]. Third, myopia, congenital and acquired glaucoma, retinoblastoma and some other disorders can change the size of the eyeball [11]. Forth, the optic canal of the sphenoid bone can be wide, normal, or narrow and specifically its orbital opening can be wide or narrow that can influence the ONSD because the sheath acts as periosteum of the sphenoid bone inside the canal [12]. All these variations might influence the accuracy of the ONSD method of intracranial pressure monitoring.

The purpose of the current research was to establish normative data of the ONSD in various locations within its intraorbital part with the help of data obtained by computer tomography (CT) technique and to analyze their possible correlations with the eyeball transverse diameter (ETD) and the optic canal diameters. We planned to measure the ONSD in several distances from the globe together with the diameters of the optic canal and the eyeball, analyze possible correlations, and recommend the most convenient approach to be used in practice in cases when ONSD is measured for the purpose of detection of elevated intracranial pressure.

## Materials and methods

### Study design and setting

In a prospective cohort study, we collected and analyzed the CT data of consecutive 400 adult patients (18+) that were admitted to the Department of Radiology at our Medical Center from Jan 2011 to February 2014. The study protocol conformed to the ethical guidelines of the 1975 Declaration of Helsinki as reflected a *priori* after approval by the institution's Helsinki committee. We examined the patients who were admitted to the Emergency Department, were referred to the CT investigation that included the head and neck region, and appeared to be neurologically and ophthalmologically healthy.

Exclusion procedure was organized in two steps. First, the patients with documented ophthalmologic, cerebral, or neurophthalmologic disorders were excluded as well as patients with injuries around the orbits. At this stage, we also checked the data of the blood tests to exclude intoxications that might affect CNS. Second, the selected patients were examined by an ophthalmologist and by a neurologist in order to exclude overlooked eye disorders or cerebral pathology. Special attention was paid in order to exclude cases with ischemic, toxic, hereditary, nutritional, or compressive neuropathies, glaucoma, cataract, etc. Therefore, four criteria were used to include a case into our study: 1) a neurologist did not find any CNS-specific pathology; 2) an ophthalmologist did find any eye/optic nerve-specific pathology; 3) CT investigation did not detect any cranial pathology or existing pathology of the optic nerve; 4) blood tests did not indicate any toxic elements that might affect the CNS.

The patient flow was as follows: from the 587 consecutive patients, 122 were excluded at the first step, 65 were excluded at the second step. The data collection was stopped when we obtained 400 healthy cases.

### Variables analyzed

1. ETD (retina to retina), 2. ONSD at 3 mm behind the globe, 3. ONSD at 10 mm behind the globe, 4. ONSD at 3 mm from the anterior lumen of the optic canal, and 5. area of the anterior lumen of the optic canal.

### Data sources and measurements

**Figure 1:**
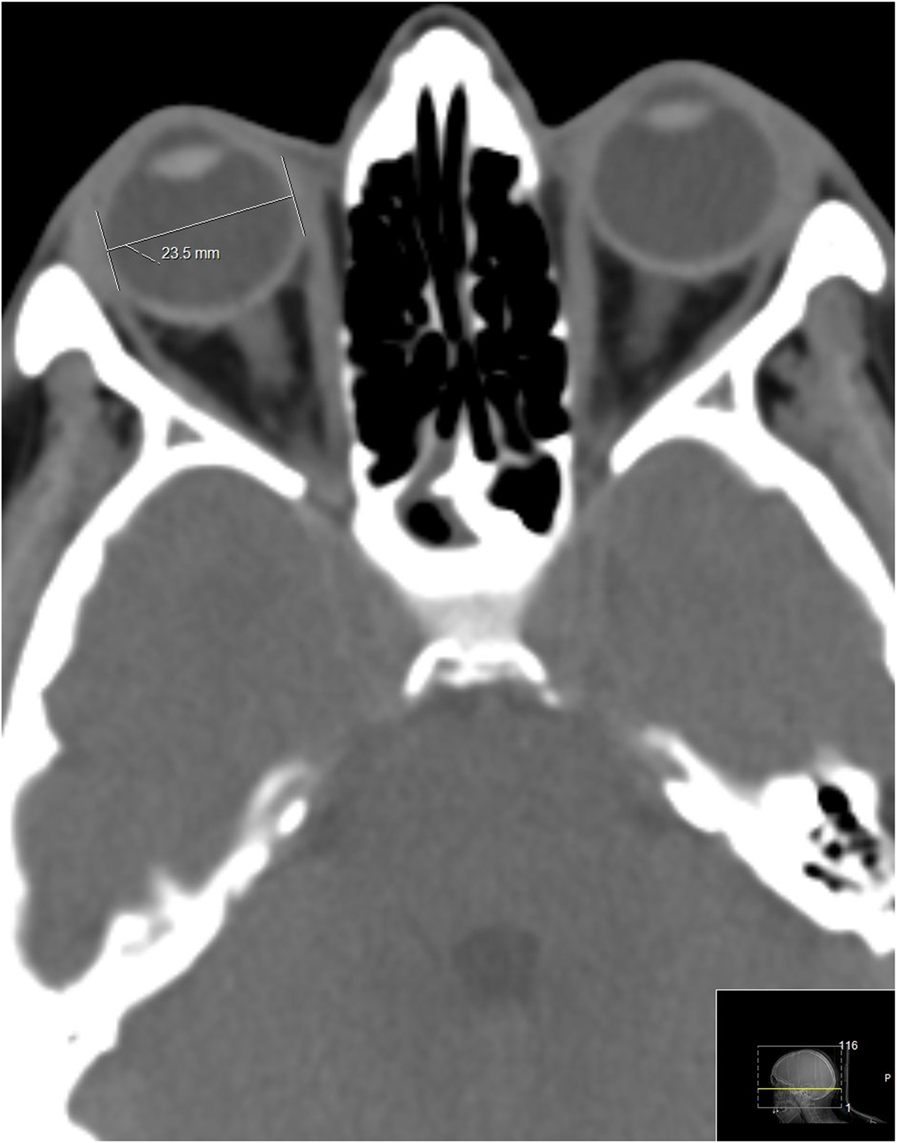
The eyeball transverse diameter and the ONSD measured at the transverse plane.

**Figure 2:**
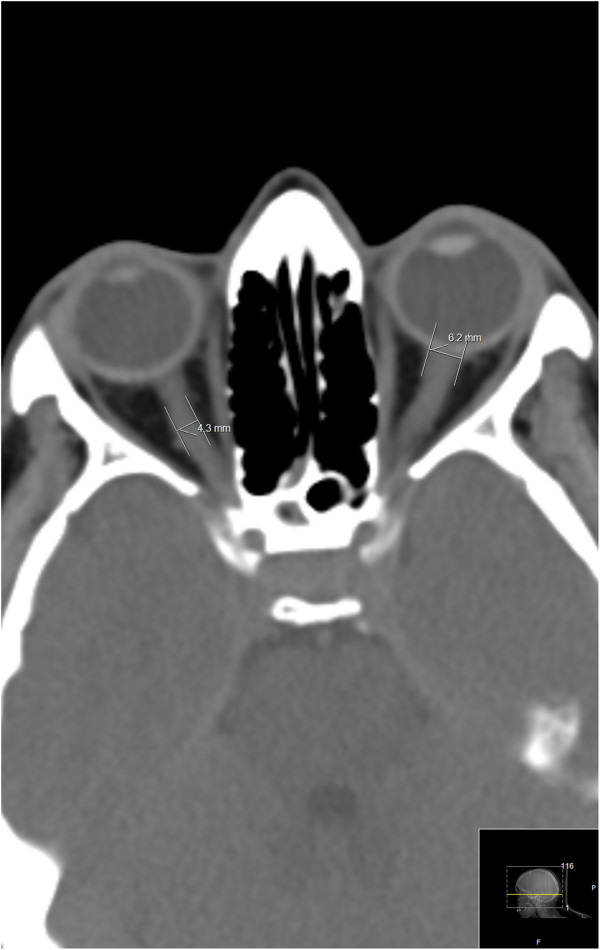
The ONSD measured at the transverse plane.

All the CT scans were obtained by the 256-slice CT scanner (Brilliance iCT, Philips Healthcare). We implemented the standard Philips protocols for head and neck imaging in all cases, single slice section 3 mm [13-15]. When the CT scans were obtained, the left and right ETD and the ONSD were measured by the computer program (Figures 1 and 2). The transverse diameter of the eyeball was chosen because the ONSD is usually measured in the transverse plain. The optic canal is rarely round in its orbital orifice; usually it is oval. That is why we measured two diameters for its orbital opening. Window parameters were: spine window, middle third; WW 60, WL 360, (sometimes abbreviated as C:60,0. W:360,0 spine), accuracy 1 pixel. All measurements were made using the same window, contrast and brightness. The error margin was expressed by means of the technical error of measurement (TEM) to calculate the intra-evaluator variability and inter-evaluator variability between two evaluators. The same equipment and methodological procedures for measurements were adopted by both evaluators.

### Analysis

Measurements of five above mentioned variables were analyzed. A within-group repeated measures experimental statistical analysis was used to test the variables. To verify the normality of the data, normal probability plots and basic descriptive statistics (mean, standard deviation (SD), min, and max) were calculated for every variable (the diameters). The data obtained from the left eyeball and the optic nerve and from the right eyeball and the nerve was compared. The correlation analysis was performed with gender and age groups (group I: 18-30; group II: 30-65; group III: 65+).

The correlation analysis was performed between the following pairs of variables: ETD to ONSD at 3 and 10 mm from the globe; ONSD converted to area at 3 mm from the lumen of the optic canal to the area of the lumen. The correlation analysis between the optic canal measurements and the proximal ONSD were performed not between their diameters but between their areas because the nerve is round and the optic canal is oval. The data were statistically evaluated by three-dimensional analysis of variance, SPSS, Standard version 17.0 (SPSS, Chicago, IL, 2007), and χ [2] criterion using 95% confidence interval. The level of significance for all analyses was set at p < 0.05.

## Results

In our cohort, there were 214 females and 186 males, age range was from 18 to 94 (mean 46). Distribution among age groups was as follows: group I (18-30) - 89; group II (30-65) - 156; group III (65+) - 155.

Altogether, 800 eyeballs, ONSD, and optic canals were measured. For the TEM calculation, two measurements were obtained from each location (n = 1600 measurements for each of three variables). The difference between the first and second measurements were then determined and the relative TEM (technical error of measurement expressed in %) was calculated to be 3.77 acceptable. For inter-evaluator TEM, it varied from 3.18 to 3.58 for different locations (acceptable).

Tables 1 and 2 present the results of the measurements. Analyzing these data it was detected that standard deviation of the mean ONSD, minimal, and maximal variations of the ONSD are the highest at 3 mm position while at 10 mm position they are the lowest. Variations of the proximal part of the ONSD that is close to the anterior opening of the optic canal are also less significant if compared with the 3 mm position. For this location, however, strong positive correlation exists between the ONS area (calculated from ONSD data) and the area of the orbital orifice of the optic canal. At the orifice itself the correlation is almost 100% (r = 0.96), and at 3 mm from the orifice it remains 0.82. In comparison, at 10 mm from the globe location r = 0.44 only.

**Table 1:** Optic nerve sheath, eyeball, and optic canal anterior (orbital) opening CT measurements (in mm)

**Distance/position**	**Right eye**			**Left eye**		
	**Mean ± SD**	**Max**	**Min**	**Mean ± SD**	**Max**	**Min**
Distal ONSD*	4.94 ± 1.51	7.5	3.5	5.17 ± 1.34	7.9	3.8
Middle ONSD**	4.35 ± 0.76	7.6	3.3	4.45 ± 0.62	5.9	3.3
Proximal ONSD***	3.84 ± 0.82	5.2	2.7	3.65 ± 0.70	4.8	2.9
Proximal ONS area	11.58 ± 1.8	21.23	5.7	10.46 ± 1.5	18	6.6
Transverse	22.822 ± 1.7	25.5	20.0	22.936 ± 1.8	25.8	19.4
Eyeball diameter						
*Optic canal anterior opening*						
Longer diameter	4.8 ± 1.8	5.8	3.5	5.0 ± 1.8	5.8	3.6
Shorter diameter	4.2 ± 1.6	4.8	2.9	4.2 ± 1.7	4.8	2.8
Area (mm^2^)	15.83 ± 2.3	19	7.2	16.49 ± 2.7	21.86	7.9

*3 mm behind the globe.

**10 mm behind the globe.

***3 mm before entering the anterior opening of the optic canal.

**Table 2:** Correlation between the optic nerve sheath, eyeball, and optic canal anterior (orbital) opening measurements (in mm)

**Correlation between**	**Right eye**	**Left eye**
	**r**	**r**
Distal ONSD*/ETD	0.74	0.69
Middle ONSD**/ETD	0.79	0.77
Proximal ONS area***/OC lumen area	0.82	0.84
ONS area at lumen/OC area at the lumen	0.96	0.95

*3 mm behind the globe.

**10 mm behind the globe.

***3 mm before entering the anterior lumen of the optic canal.

ETD – eyeball transverse diameter.

OC – optic canal.

Analyzing further the obtained results, we paid attention that ONSD taken from the middle section of the intraorbital part of the optic nerve correlates with the ETD of the eyeball and that this correlation can be presented as an index. This index is calculated as ONSD divided by the transverse diameter of the eyeball (ONSD/ETD) and is presented in the Table 3 as 0.19 with standard deviation of 0.01-0.02.

**Table 3:** The nerve/eye index in healthy adults

**ONSD/ETD index**	**Right eye**	**Left eye**
Average	0.19 ± 0.01	0.19 ± 0.02
Max	0.26	0.26
Min	0.15	0.15

The index is calculated as ONSD taken from the middle part of the intraorbital path of the optic nerve divided by the transverse diameter of the eyeball (ONSD/ETD).

We did not find statistically significant differences correlated with gender of the patients (p = 0.15), and their age (I vs. II, p = 0.25; I vs. III, p = 0.09; II vs. III, p = 0.36). In our cases, measurements taken from the right eyeball and optic nerve were slightly smaller than the left side measurements but this difference is also statistically insignificant (p = 0.44).

## Discussion

The pathophysiology of optic nerve sheath enlargement as a result of intracranial hypertension has been established well already [6,7,16]. The ONSD technique itself is not perfected yet and some improvements might be suggested. Analyzing the obtained data, we believe that the 3 mm distance from the globe is not the ideal location to measure ONSD for intracranial pressure monitoring. We cannot ignore constant physiological tremor, slow drifts, flicking movements, tracking movements, smooth pursuits, saccades, and other eye movements [17,18]. Whatever method is used for the ONSD measurement – CT, MRI, or ultrasound – images are taken from a constantly moving object even when a patient is given instruction to look straight forward, and even when the eyes are closed. Currently, the quantitative estimate of how the movements of the eyeball change shape and size of the bulging dura mater region is lacking. We cannot recommend measuring the ONSD close to the globe until this question is clarified. In addition to that, the enlargement of ONSD behind the globe was also found in papilledema, optic nerve lesions, optic atrophy, and endocrine orbitopathy [19,20].

We did not find statistically significant differences in ONSD correlated with age. The optic nerves experience the age-dependent nerve fiber loss as any other nerve in the human body. However, while total axon count in the optic nerve decreases with age, mean axon diameter increases with age [21]. At the same time, the thickness of dura mater increases with age [22]. While all these processes take place simultaneously, we might suggest that the ONSD remains approximately the same during a lifetime.

The size of the eyeball correlates with the ONSD. This fact can be used to our advantage. The optic nerve/eyeball diameter index is much less variable variance than ONSD and could be used for intracranial pressure monitoring with more precise results.

Movements of the eyeball can change ONSD close to the globe therefore 3 mm distance from the globe is not an ideal location to measure ONSD to monitor intracranial pressure. If ONSD is measured close to the orbital orifice of the optic canal, the measurements can be influences by the correlation between dimensions of the ONS and the optic canal. If the canal itself and especially if its anterior opening is wide or narrow, the ONSD measurement will correlate with it. Therefore, the proximal location is also not ideal for measuring the ONSD for intracranial pressure purposes. The middle part of the intraorbital optic nerve route experience less variations in size in normal healthy people. We do not dictate that ONSD should be measured at 10 mm from the globe sharp; the intraorbital part of the optic nerve varies in length (usually from 1.5 to 2.4 cm) and the ONSD can be measured at 8 mm or 12 mm from the globe but definitely not at 3 mm location. In any case, for the most precise detecting of the elevated intracranial pressure, we recommend to use the optic nerve/eyeball diameter index. This index is calculated as ONSD taken from the middle part of the intraorbital path of the optic nerve divided by the transverse diameter of the eyeball (ONSD/ETD). While standard deviation of the ONSD measurements varies from 0.62 to 1.51 at various locations, the standard deviation of the ONSD/ETD index is 0.01-0.02 that insures very precise normative data. From three eyeball diameters, we selected the ETD because the majority of the authors writing on ONSD technique for intracranial pressure monitoring measure ONSD in the transverse plain and because anterior-to-posterior eyeball diameter varies in cases of myopia, emmetropia, and hypermetropia significantly.

### Limitations of the research

All the CT scans were obtained by the 256-slice CT Philips scanner. It might be possible that scanners of different trademarks could provide slightly different results of measurements as well as MRI or sonography evaluation.

### Generalisability

External validity of the study results is based on recent efforts in standardization of CT nomenclature and protocols for various CT scanner manufacturers (GE, Philips, Toshiba, Hitachi, Siemens). All these scanner manufacturers provide features to automatically initiate a prescribed axial, helical or dynamic scan when a threshold level of contrast enhancement is reached at a specified region of interest (in our case, the orbit and the optic canal) [23].

## Conclusion

In healthy persons, the ONSD varies from 3.65 mm to 5.17 mm in different locations within the intraorbital space with no significant difference between sexes and age groups. More precise results can be obtained through the calculation of an index when ONSD is divided by the ETD of the eyeball. In healthy subjects, the ONSD/ETD index equals 0.19. When the ONSD is measured for intracranial pressure monitoring, the most stable results can be obtained if the diameter is measured 10 mm from the globe. These data might serve as a normative database when ONSD technique is used for intracranial pressure monitoring at emergency departments and in general neurological practice.

## Competing interests

The authors declare that they have no financial and non-financial conflict of interest.

## Authors’ contributions

MV – study concept, study design, analysis of the data, manuscript draft, manuscript final version; PG and IB – collection of the data, data analysis. All authors read and approved the final manuscript.
